# Dynamics and Control of a Novel Hybrid Legged Robot with Temporary Flight Capabilities

**DOI:** 10.3390/biomimetics11050328

**Published:** 2026-05-08

**Authors:** Emir Kutluay, Oğuzhan Gültekin, Yiğit Yazıcıoğlu

**Affiliations:** 1 Department of Mechanical Engineering, Hacettepe University, 06800 Ankara, Türkiye; kutluay@hacettepe.edu.tr; 2 Department of Mechanical Engineering, Middle East Technical University, 06800 Ankara, Türkiye; yigit@metu.edu.tr

**Keywords:** flying RHex robot, legged robots, temporary flight, flying legged robots, model linearization, modern control

## Abstract

In this study, a novel flying legged robot configuration with enhanced obstacle-crossing capability is introduced. Legged robots, especially RHex robots, already possess high obstacle-crossing capability; however, the obstacle size that can be overcome is directly dependent on the leg length. Although stair climbing–descending, obstacle course and inclined surface algorithms have been studied for the RHex robot, flight capability has not been explored. In this study, this improvement is achieved with minimal impact on the RHex’s design by adding just a thruster as an additional propulsion system to propel the robot into flight. The attitude control is realized using the mass actuation of the robot legs, similar to how animals like lizards and cats utilize their limbs or tails as inertial appendages to stabilize their body pitch during mid-air maneuvers. Instead of direct and complete flight control, the aim was a temporary flight similar to obstacle-clearing flights of chickens. Hence, a nonlinear 2D model is developed to investigate the kinematics and dynamics of the RHex robot. Equations of motion are derived, linearized and used in a state feedback regulator design; the regulator is also expanded for reference tracking.

## 1. Introduction

This study demonstrates the feasibility of a flying legged robot, which employs only a thruster in addition to its original configuration and whose attitude control is achieved by reaction torques and forces generated by its legs.

The chosen platform, RHex, is a six-legged robot with a high degree of mobility, inspired by cockroach locomotion for robust traversal of uneven terrain [1]. The legs of RHex are capable of full rotation, thereby eliminating the need to retract or bend the legs to shorten their total length when stepping forward. To achieve an appropriate walking and running sequence, the stance phase of the leg is slowed down while the swing phase is accelerated. Due to this movement pattern, three legs always remain in contact with the ground during walking. These characteristics significantly enhance RHex’s speed, maneuverability, and stability. Furthermore, the RHex platform has been extensively researched and worked upon, with a number of studies conducted on its ability to navigate stairs [2] and move over challenging surface conditions [3]. Additionally, a hybridization effort has been undertaken to enable it to move in water [4].

In the broader context of mobile robotics, navigating environments with ground obstacles often relies on sophisticated compliance algorithms and complex kinematics, such as the variable admittance-control strategies employed in wheeled mobile manipulators [5]. However, for purely ground-based robots, physical barriers that significantly exceed the vehicle’s structural dimensions remain a strict limitation, which strongly motivates the integration of aerial locomotion. Hybrid robots are created by combining multiple locomotion mechanisms [6]. There are studies on hybridizing terrestrial robots to improve their obstacle-crossing capabilities [7,8,9,10,11]. This combination can sometimes involve utilizing the same type of locomotion with different limbs (e.g., wheels or tracks that can transform into legs) or transitioning between different types of locomotion (e.g., robots that can both fly and move on land).

Hybrid motion has been classified into two categories by Russo and Ceccarelli: reconfigurable and non-configurable motions. In reconfigurable motions, the same mechatronic system is utilized for both modes of movement [6]. This usage can vary, such as in the FSTAR [12], where the same motor is employed to control both the wheels during terrestrial movement and the propellers during aerial movement, or in the HyTAQ robot [13], where propellers generate force in the desired direction during terrestrial movement, and the cylindrical cage-shaped body is propelled along the ground by this force. In non-configurable movements, the structures belonging to the two locomotions are separated. Examples of this group include the HyLMoR [14] and DUCK [15] robots. The HyLMoR robot has a quadrotor structure for flying and a four-legged structure for walking on land. The DUCK robot also has a quadrotor structure for flying but uses underactuated bipedal legs for walking on land. The DUCK robot employs its propellers during terrestrial movement unless it is moving downhill.

**Figure 1:**
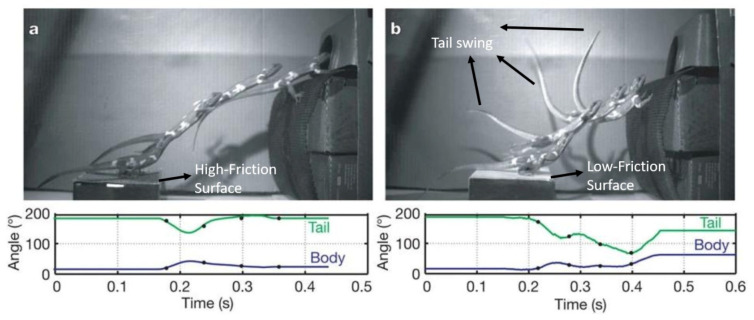
Mid-air body reorientation and pitch control of a lizard using tail inertia. (**a**) A stable leap from a high-friction surface with minimal attitude perturbation. (**b**) A leap from a low-friction (slippery) surface, inducing a pitch-down perturbation, which is actively counteracted by a 110° upward tail swing to reorient the body mid-air. The leg reaction torque principle utilized in our robot design exhibits a biomimetic similarity to the stabilization mechanism provided by the lizard’s tail in this figure.

In this study, a reconfigurable type of hybridization is presented. It is considered reconfigurable because the RHex robot’s control in flight is achieved using reaction torques of its legs. This situation can be likened to a tightrope walker who uses their arms and legs to maintain balance or to cats, lizards, and certain insects, which utilize their limbs or tails as inertial appendages to maintain orientation and stabilize their body pitch during mid-air maneuvers. For instance, biomechanical studies demonstrate that praying mantises achieve precise mid-air attitude control by exchanging angular momentum between their rapidly rotating legs and abdomen [16]. Similarly, lizards have been shown to employ tail-assisted pitch control, where measured swings of their tails redirect angular momentum to counteract unexpected body rotations in the sagittal plane [17]. As illustrated in the experimental sequence in Figure 1, when a lizard leaps from a low-friction surface, the resulting slip induces an unexpected forward pitch perturbation. To counteract this destabilizing rotation, the animal actively swings its tail upward, effectively using inertial forces to reorient its main body in mid-air and ensure a stable trajectory. In the proposed flying RHex configuration, the robot’s legs perform exactly the same function: by applying controlled torques to the rotating legs, reaction torques and forces are generated on the body, enabling pitch stabilization and thrust-vector redirection during temporary flight.

As illustrated in the experimental sequence in Figure 1, lizards provide a compelling biological example of this stabilization mechanism. When leaping from a low-friction surface, the resulting slip induces a sudden forward pitch perturbation. To counteract this destabilization, the lizard actively swings its tail upward, utilizing inertial forces to reorient its body in mid-air and maintain a stable trajectory. Inspired by this tail-assisted pitch control, the proposed flying RHex configuration relies on its rotating legs to perform the exact same function. By applying controlled torques to the legs, the robot generates the necessary reaction torques to stabilize its pitch. With the attitude control successfully managed by these leg-induced inertial forces, the system only requires an independent vertical propulsion source to achieve temporary flight.

The vertical force required to support body weight is maintained by adding only a hypothetical thruster. This thruster is treated as a force generator in only one direction with no net yaw moment applied to the system. Hence, the RHex robot is provided with a force for vertical movement, and it controls the angular position of its body, as well as the direction of the thrust vector of the thruster fixed to its body, using the reaction torques of its legs. In this way, the robot can be flown in the desired direction.

We consider the robot inputs as the torque applied to the legs and the force applied to the robot body by the body-fixed thruster. Torque control on a reaction wheel is detailed in the works of Carrara and Kuga [18]. Attitude control of the body using the reaction torques and forces generated by the legs is closely analogous to how it is done with reaction wheels in satellites, but there are several key differences. The most significant difference stems from the asymmetrical structure of the legs, which act as unbalanced masses. When the angle of the legs is changed, there is also a change in the center of mass of the total system, whereas such a situation does not occur with reaction wheels, since they are symmetrical. Another important difference is seen in momentum unloading [19]. In satellite attitude control, accelerating the reaction wheels causes momentum gain, as long as they are energized, and this momentum is conserved. However, the legs’ interaction with air is quite strong due to their curvy shape, and an additional reaction torque can be obtained in relation to their rotational speed. As a result, a self-momentum unloading effect is generated. For this reason, although air friction is useful, aerodynamic drag of the legs is neglected for simplification, and this phenomenon is not modeled.

Our work presents a robot that uses its legs for stabilization in the air, in contrast to how the Leonardo robot uses a quadrotor flight system for stabilization on land [20]. The RHex robot, which uses its unique leg configuration and a fixed thruster to maintain balance in flight, demonstrates a novel method to achieve short-duration aerial maneuvers. With the addition of flight capability, the RHex robot can function through a larger operating envelope and overcome obstacles that were otherwise unsurpassable.

## 2. Methods

### 2.1. Kinematics

The RHex robot is a hexapedal robot with a circular gait mechanism. In this study, only a 2D and simplified model of the robot with only four legs is considered. This 2D simplified consideration is strategically adopted to establish a fundamental dynamics baseline of a flying RHex robot and to isolate analysis of the longitudinal stabilization mechanisms. The yaw motion of the robot can be either decoupled from the pitch motion by utilizing a dual-propeller ducted fan system or controlled in a coupled manner using two counter-rotating ducted fans. To maintain the simplicity of this preliminary study, yaw control was excluded. Regarding the roll motion, although it is known to be divergent during the highlighted temporary flight duration, it was assumed that surmounting obstacles despite potential deviations is preferable to failing to clear them at all. Furthermore, while three-axis control could be achieved by introducing slight angles to the legs, we preferred to maintain the original terrestrial structure of the RHex robot without any modifications. Thus, the robot body has 3-DoF: horizontal translation, vertical translation and pitch rotation around the normal axis to the plane.

**Figure 2:**
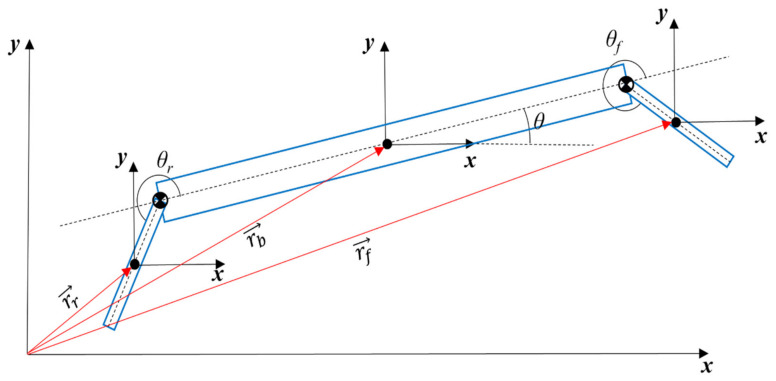
Simplified RHex robot with body position vectors.

In this simplified model, each leg contributes an additional DoF as rotation around the joint axis while the left and right legs are controlled symmetrically. This model is shown in Figure 2. The body position vectors with respect to an Earth-fixed coordinate frame are also presented in the figure. Here, r,v and a stand for body center of mass position, velocity and acceleration vectors, respectively. θ, ω, and α represent the body pitch angle, angular velocity and angular acceleration, respectively. Rear and front leg angles are defined with respect to the body’s horizontal forward axis. Subscripts b, l, f, and r are used for body, leg, front-leg-, and rear-leg-related variables, and e is used to represent the Earth-fixed reference frame; for instance, ${\vec{\omega }}_{b/e}$ denotes angular velocity of the body with respect to the Earth-fixed reference frame. T, W  and F are used for thrust force applied by the thruster, weights and the forces. $T_{f}$ and $T_{r}$ symbolize the front and rear leg torques, respectively. $F_{rby}$, $F_{fby}$, $F_{rbx}$ and $F_{fbx}$ are the forces applied by the rear leg to the body in the y-direction, the front leg to the body in the y-direction, the rear leg to the body in the x-direction, and the front leg to the body in the x-direction, respectively. Parameters and their symbols are given in Table 1 with their values.

Body position vectors are given in Equations (1)–(3).

(1) r→b=xl^+yJ^

(2) r→f=xl^+yJ^+Lfcos θ l^+Lfsin θ J^+Rfcos θ+θfl^+Rfsin θ+θfJ^ 

(3) r→r=xl^+yJ^+Lrcos(θ+π)l^+Lrsin(θ+π)j^+Rrcosθ+θrl^+Rrsinθ+θrJ^

Body velocity and acceleration vectors are obtained from body position vectors by differentiation. Body angular velocity vectors are given in Equations (4)–(6).

(4) ω→b/e=θ˙k^

(5) ω→f/e=ω→b/e+ω→f/b=θ˙k^+θ˙fk^

(6) ω→r/e=ω→b/e+ω→r/b=θ˙k^+θ˙rk^

Body angular acceleration vectors are given in Equations (7)–(9).

(7) α→b/e=θ¨k^

(8) α→f/e=α→b/e+α→f/b+ω→b/e×ω→f/b=θ¨k^+θ¨fk^

(9) α→r/e=α→b/e+α→r/b+ω→b/e×ω→r/b=θ¨k^+θ¨rk^

Coriolis terms shown in Equations (8) and (9) vanish due to the simplified 2D model.

### 2.2. Derivation of EoMs

**Figure 3:**
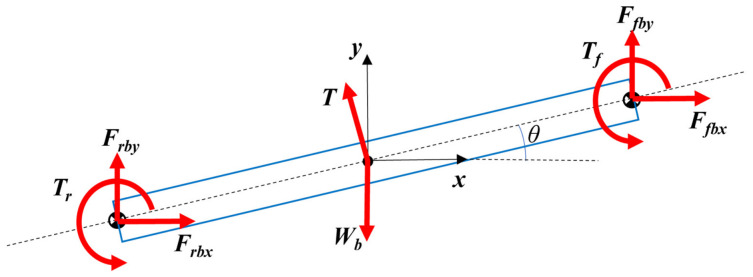
Free-body diagram of robot body.

Two constraint forces $F_{fb}$ and $F_{rb}$, and the reaction torques of the leg motors $T_{f}$ and $T_{r}$ are applied to the main body for each one of the legs. The external forces acting on the body are pure forces without a couple, which are generated by the hypothetical thruster and the gravitational force. The free-body diagram of the body is given in Figure 3.

Scalar force and moment equations for the body are given in Equations (10)–(12):

(10) $$\sum F_{x}=\;F_{rbx}+F_{fbx}+Tcos\left(\frac{\pi }{2}+\theta \right)=M_{b}a_{bx}\;$$

(11) $$\sum F_{y}=F_{rby}+F_{fby}+Tsin\left(\frac{\pi }{2}+\theta \right)-W_{b}=M_{b}a_{by}\;$$

(12) $$\sum M_{z}=T_{f}+T_{r}-L_{f}\sin\left(\theta \right)F_{fbx}+L_{f}\cos\left(\theta \right)F_{fby}+\;L_{r}\sin\left(\theta \right)F_{rbx}-L_{r}\cos\left(\theta \right)F_{rby}=I_{b}\alpha_{b}\;$$

**Figure 4:**
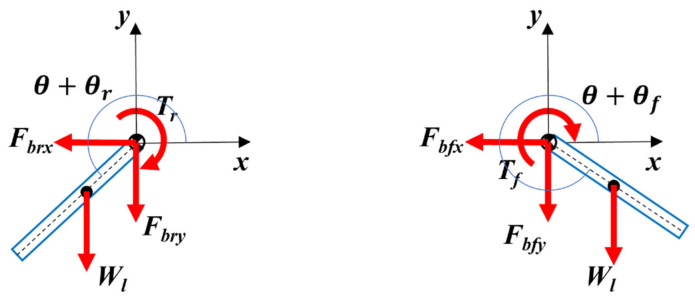
Free-body diagram of robot legs.

Figure 4 provides the rear and front legs’ free-body diagram.

Scalar force and moment equations for the legs are given in Equations (13)–(18).

(13) $$-F_{bfx}=M_{l}a_{fx}$$

(14) $$-F_{bfy}-W_{l}=M_{l}a_{fy}$$

(15) $$-T_{f}+Rsin\left(2\pi -\theta -\theta_{f}\right)F_{bfx}+Rcos\left(2\pi -\theta -\theta_{f}\right)F_{bfy}=I_{l}\alpha_{f}$$

(16) $$-F_{brx}=M_{l}a_{rx}$$

(17) $$-F_{bry}-W_{l}=M_{l}a_{ry}$$

(18) $$-T_{r}-Rsin\left(\theta +\theta_{r}\right)F_{brx}+Rcos\left(\theta +\theta_{r}\right)F_{bry}=I_{l}\alpha_{r}$$

The constraint forces $F_{bfx},\;F_{bfy},\;F_{brx},\;F_{bry}$ can be found in terms of other variables in Equations (13), (14), (16) and (17) and substituted into the Equations (10)–(12), (15) and (18), yielding five equations of motion. The detailed equations are given in Appendix A. If the EoMs are reorganized to have all acceleration variables with their multiplicative terms on the left-hand side and all other variables on the right-hand side, the resultant equations can be rewritten in matrix form, as in Equation (19). The K matrix shown in Equation (19) is known as the mass matrix, and it is a matrix function of only position variables. All remaining terms are collected in vector l. The acceleration variables may be obtained by inverting the matrix K and left-multiplying with the l vector.

(19) $$[K{]}_{5\times 5}\times \left\{\ddot{x}\left(t\right)\;\ddot{y}\left(t\right)\;\ddot{\theta }\left(t\right)\;{\ddot{\theta }}_{f}\left(t\right)\;{\ddot{\theta }}_{r}\left(t\right)\;\right\}={\left\{l\right\}}_{5\times 1}$$

### 2.3. Validation of EoMs

**Figure 5:**
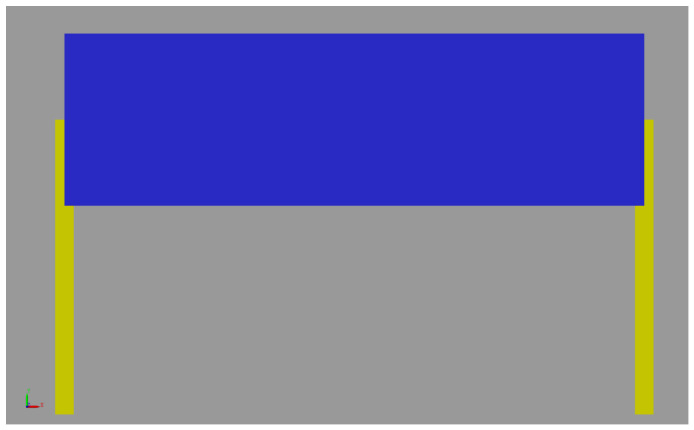
Simplified RHex robot Simscape^®^ model.

In this section, the EoMs found in the previous section are compared with the Simscape^®^ v23.2 multibody dynamics software. In the previous section, the K matrix and l vector are found in terms of position and velocity variables with inputs and system parameters. The calculated acceleration variables are integrated to obtain the velocities and positions of the body and legs, and this process is repeated at each step. In order to verify the equations, a multibody model of the system with the same parameter set is designed using the Simscape^®^ v23.2 tool of Matlab (R2023b). The Simscape^®^ model is given in Figure 5.

**Figure 6:**
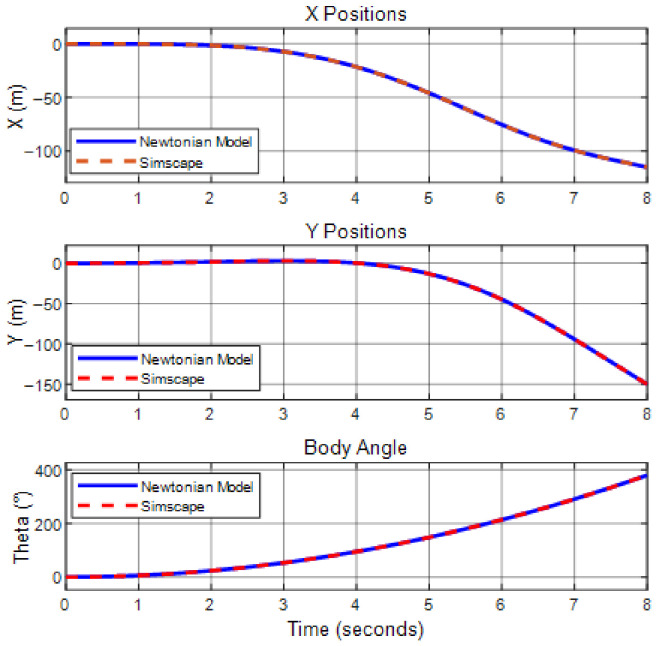
Simscape^®^ and mathematical model comparisons for body positions and pitch angle.

**Figure 7:**
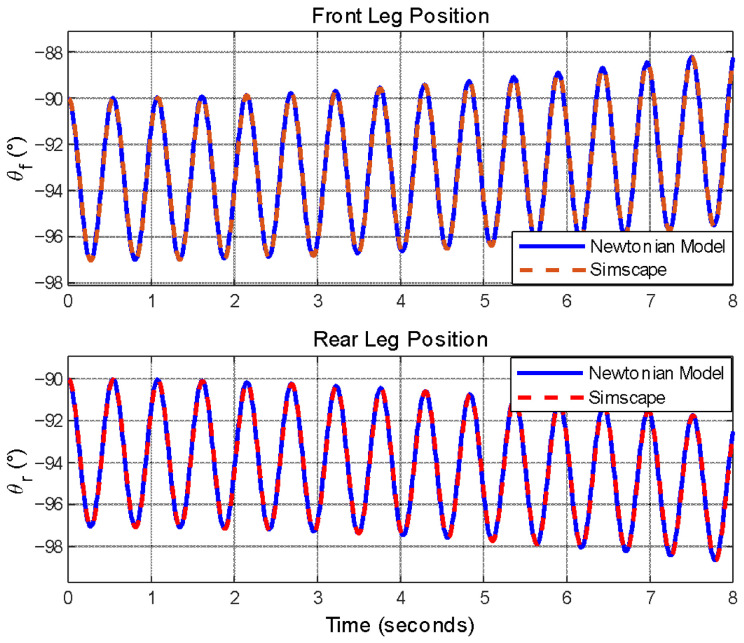
Simcape^®^ and mathematical model comparisons for leg angles.

The open-loop step responses of both models have been compared and given in Figure 6 and Figure 7. The absolute and relative errors between the two models have also been examined, with the absolute error being in the order of $10^{-7}$ and the relative error in the order of $10^{-8}$. These errors are considered natural since they are below the resolution used for defining parameters such as inertia, mass, and lengths. Therefore, these errors do not pose any hindrance to the continuation of this study.

When examining the system responses provided in Figure 6 and Figure 7, it is observed that both systems exhibit identical responses to the same inputs. This kind of analysis offers insight into the accuracy of the equations. These equations will next be linearized and used for designing a linear controller. In this section, it has been demonstrated that the mathematical model and the Simscape^®^ model can be used interchangeably, allowing the linearization and the controllers will be tested in Simscape^®^ in the subsequent sections. This will provide the advantage of conducting the analyses visually.

### 2.4. Linearization

A linear state-feedback controller architecture is used for the flight control of the robot. The first-order Taylor series expansion method is followed in the linearization process. Position, velocity and acceleration variables and inputs are defined in Equation (20) for brevity.

(20) r^=xt yt θt θft θrt ,  v^=x˙t y˙t θ˙t θ˙ft θ˙rt ,                                   a^=x¨t y¨t θ¨t θ¨ft θ¨rt  ,  u^=T Tf Tr 

Equation (20) is rewritten as given in Equation (21).

(21) a^=fr^,v^,u^=[K]5×5−1×{l}5×1

The expansion of Equation (21) is given in Equation (22).

(22) a^=v^˙=fr^,v^,u^≈a^0+∂f∂r^|r^0,v^0,u^0r^−r^0+∂f∂v^|r^0,,v^0,u^0v^−v^0+∂f∂u^|r^0,v^0,u^0u^−u^0 

The difference in states is defined as in Equation (23); Equation (24) is obtained by direct substitution.

(23) δr^=r^−r^0,  δv^=v^−v^0,  δv^˙=v^˙−v^˙0,  δu^=u^−u^0

(24) δv^˙≈∂f∂r^|r^0,v^0,u^0δr^+∂f∂v^|r^0,v^0,u^0δv^+∂f∂u^|r^0,v^0,u^0δu^

Since the mass matrix K is only a function of position variables and the l column vector is a function of position, velocity and input variables, one can use the derivative of the inverse formula [21] and obtain Equation (25).

(25) δv^˙=−K−1∂K∂r^K−1l+K−1∂l∂r^|Hδr^+K−1∂l∂v^|Hδv^+K−1∂l∂u^|Hδu^ 

where H stands for linearization points $p_{0},v_{0},u_{0}$.

$A_{1},\;A_{2}$ and $B_{1}$ matrices are defined for brevity as shown in Equations (26)–(28).

(26) A1=−K−1∂K∂r^K−1l+K−1∂l∂r^|H

(27) A2=K−1∂l∂v^|H

(28) B1=K−1∂l∂u^|H

Here, ∂l∂r^ and ∂l∂v^ are the Jacobians of the l vector with respect to the position and velocity states, respectively. On the other hand, $\frac{\partial K}{\partial p}$ is the partial derivative of a 5 × 5 matrix with respect to position states shown in Equation (29).

(29) ∂K∂r^ijk=∂Kij∂r^k,  i,j,k=1, 2, 3, 4, 5

Here, i,j and k indices indicate the row, column and layer numbers. Following the definitions, the system can be written in continuous state-space form as in Equation (30).

(30) x˙=δr^˙ δv^˙ =0 I A1 A2 {r^ v^ }+0 B1 {δu^}

The equilibrium point for linearization is selected as the hover condition, and initial values of the states are presented in Table 2. Initial body position and velocity are accepted as zero as well as initial leg torques. The initial magnitude of the thrust vector is taken to be equal to the total weight of the system. The legs are accepted as pointing downward and in rest, so the leg angles are 270° with no rotational speed. Body parameters are assumed to be the same as the nonlinear model given in Table 1.

Resultant state space matrices are given in Equations (A6) and (A7) in the Appendix A.

When controllability is checked for the obtained system and input dynamic matrices, the system is found to be fully state controllable, so a full state feedback controller can be designed in the next section.

**Figure 8:**
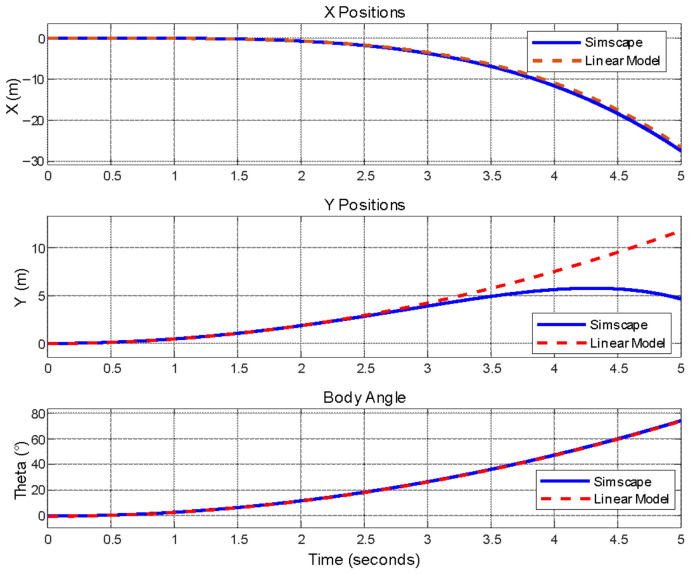
Linear and Simscape^®^ model comparisons for body positions and pitch angle.

Following the linearization, the linear model is also validated with the Simscape^®^ model. The input magnitudes applied to models are kept within linear limits. Simscape^®^ model responses and linear model responses for body translational and angular positions are compared. The results are given in Figure 8.

The body pitch angle and longitudinal position responses of both models show satisfactory agreement, as the shared components—such as inertia, leg torque inputs, and thrust force—dominate the dynamics, while the nonlinear model’s additional considerations, like center of mass variations and thrust force fluctuations, have negligible effects in the analyzed scenarios.

The vertical position of the robot is well matched while the body pitch angle is less than 20°, although the response separates with increasing body rotation due to nonlinear effects.

### 2.5. Controller and Results

A linear state feedback controller configuration is preferred in this study. The closed-loop system specifications are determined to ensure adequate performance in flight, since the robot is only required to fly temporarily. The robot is expected to track horizontal and vertical velocity reference step inputs as it is used in many studies in drone control [22]. A settling time of 6 s and maximum overshoot in velocity of 5% are targeted. All of the state variables are assumed to be measured.

Desired dominant second-order poles and arbitrarily selected remaining poles, which are placed away from the dominant poles, are given in Equation (31).

(31)   s={−0.67−0.70i,−0.67+0.70i,−3.0,−4.0,−4.0,−5.0,−5.0−6.0−6.0−7.0}

The resultant gain matrix is calculated as given in Equation (32).

(32) $$K_{f}=\left[\begin{array}{cccccccccc} 58.3 & 89.3 & -1244 & 142 & 129 & 123 & 34.4 & -530 & 4.26 & 2.05\\ -5.24 & -0.53 & 127.0 & 4.41 & -19.5 & -11.8 & -0.09 & 63.3 & -3.27 & -0.7\\ 0.995 & 0.475 & 37.4 & -10.1 & 13.8 & -1.88 & 0.078 & 25.7 & -0.484 & -3.1 \end{array}\right]$$

Leg torques are converted to units of mNm to express the second and third rows of $K_{f}$ more rigorously.

### 2.6. Model Assumptions, Validity and Practical Implementation Considerations

The 2D simplified model intentionally neglects yaw dynamics (addressed in future work by dual counter-rotating ducted fans), aerodynamic drag on legs and body, and joint friction. These assumptions were adopted to isolate the core longitudinal stabilization mechanisms and establish a fundamental dynamics baseline. Neglecting aerodynamic drag slightly overestimates the required leg torques for pitch control; in reality, drag provides a beneficial self-momentum-unloading effect (already noted in Section 1) [23,24] but also introduces additional damping that would improve stability margins [25,26]. The 2D assumption limits direct applicability to 3D flight but remains valid for preliminary feasibility of temporary straight-line or low-yaw maneuvers.

Task-oriented validations (e.g., short-duration obstacle-clearing flights and robustness to external disturbances or parameter uncertainty) fall outside the scope of the present foundational study. These analyses, including Monte-Carlo disturbance tests and representative maneuver simulations, were performed in the complementary study by Başer et al. [27] in their thesis and will be presented in a separate forthcoming article publication.

## 3. Results

**Figure 9:**
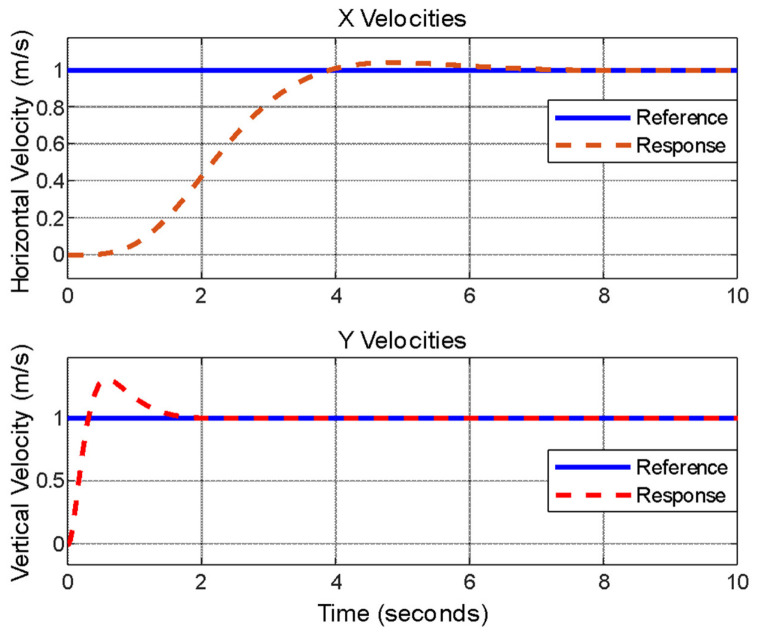
Simultaneous unit step input reference tracking.

The designed controller is tested with simultaneous 1 m/s step inputs. Responses are presented in Figure 9.

Desired overshoot and settling time requirements are satisfied. Time domain metrics are presented in Table 3.

**Figure 10:**
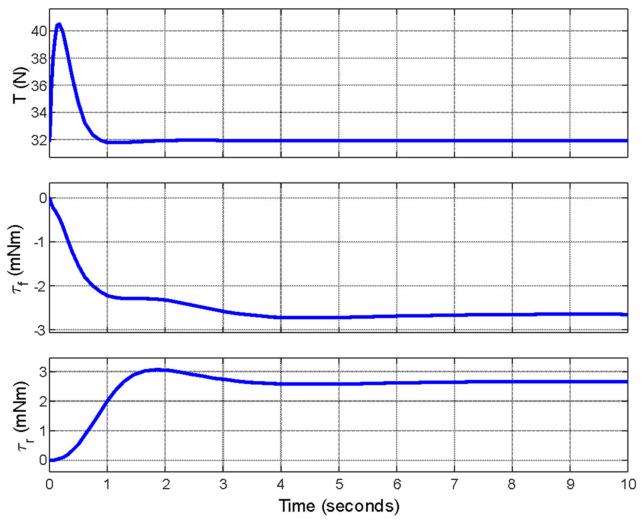
Thrust force (**top**), front (**middle**) and rear (**bottom**) leg actuator efforts.

Actuator efforts for these inputs and responses are given in Figure 10.

A thrust input of approximately 40.5 N is required for this maneuver, and a thruster with higher capacity can be selected since for many electrically powered aerial drones, a thrust capacity of twice the vehicle’s weight is feasible [28]. Nevertheless, the thrust (≈40.5 N) and leg-torque values reported in this study are based on the idealized parameters of the preliminary 2D model and serve to demonstrate basic feasibility. A parallel engineering analysis conducted by Başer et al. [27] performed detailed component selection (ducted fans, motors, and power systems) and determined that realistic off-the-shelf thrusters and structural reinforcements would approximately result in one and a half of the required thrust magnitude and overall vehicle mass while remaining within practical weight and energy budgets for short-duration flights. A comparison between idealized model parameters and realistic hardware parameters is given in Table 4. Those findings will be reported separately; the present work intentionally isolates the novel leg-based attitude-control concept without hardware-specific optimization.

**Figure 11:**
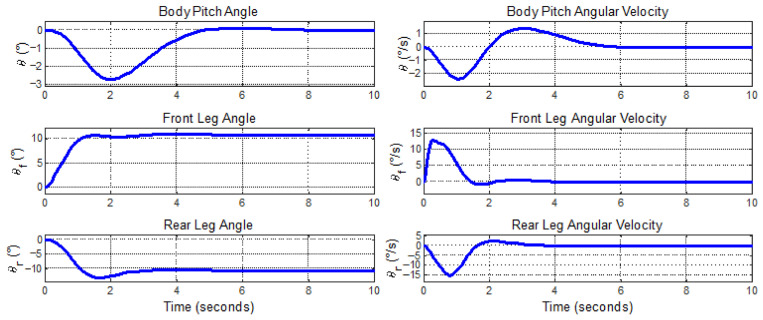
Body pitch (**top**), front leg (**middle**) and rear leg (**bottom**) angles and angular velocities.

Respective leg motions to unit step velocity inputs in horizontal and vertical axes are given in Figure 11.

The body pitch and leg rotation angles converge to zero since the model does not include drag effects. With the air friction force modeled, the body pitch angle and the leg rotation angles will be stabilized at non-zero values to overcome air friction.

In this section, a linear state-feedback controller based on pole placement around the hover condition was deliberately chosen as the simplest method to demonstrate the feasibility of the novel leg-based attitude-control concept. While more advanced techniques such as LQR, MPC, or nonlinear control could further improve performance and explicitly handle input saturation, they are beyond the scope of this foundational study. Future work will explore these methods; the present controller successfully proves the viability of the concept with the desired settling time and overshoot specifications with respect to applied simultaneous step inputs. Due to non-minimum phase zeros in the closed-loop system, the robot has a 1-s delay time. The required thrust force is found to be acceptable. The leg torques are approximately 6 mNm for a unit step response, which is acceptable for hypothetical actuators. On the other hand, this might be a problem since these motors should be selected to actuate walking motion. This requires oversized actuators for flight control, and it is hard to control those motors for small actuation torques, so an optimization will be required in production. After all investigations, the control of the robot is successfully achieved, and the 2D simplified model is shown to be hybridizable with temporary flight capability.

This study is explicitly a preliminary feasibility investigation based on nonlinear modeling and Simscape validation only. No physical prototype or hardware experiments were performed. Practical implementation challenges include actuator sizing, power budgeting for short-duration flights, and integration of the body-fixed thruster without compromising terrestrial mobility. These engineering aspects are addressed in detail in the complementary study by Başer et al. [27], which will be reported separately.

## 4. Conclusions

In this study, a novel flying legged robot configuration is introduced. A typical RHex robot, which employs six legs for terrestrial locomotion, is chosen as the hybrid platform candidate. Temporary flight capability is proposed by utilizing a hypothetical thruster with no net yaw moment. In order to realize control during flight, no modifications are made to the original configuration of the robot, and instead reaction torques of the legs are exploited.

Table 5 compares the proposed leg-based attitude control with representative hybrid flying legged robots reported in the literature. The key advantages of the present approach are minimal added mass (only a single fixed thruster), preservation of the original RHex terrestrial structure, and utilization of existing leg actuators for flight stabilization—features not simultaneously achieved in prior designs such as HyTAQ, DUCK, Leonardo and FSTAR robots.

The flight model of the proposed flying legged robot is developed with multibody dynamics software Simscape^®^ v23.2. A nonlinear 2D dynamic model is also formulated to develop the controller. No joint friction or air friction is included in the model for simplification. The dynamic model is validated by comparing with the multibody flight model. The nonlinear 2D dynamic model is linearized by computing the partial derivatives of the symbolic mass matrix for Taylor series expansion first and then substituting the operating conditions and inverting the result.

Then using the linearized 2D model, a linear state feedback regulator is designed and analyzed for attitude-stabilization performance. Subsequently, the regulator is extended to develop a servo controller, and the reference tracking capability of the hybrid structure is investigated. The designed controller is implemented in the multibody model, and the feasibility of the novel flying legged robot configuration is demonstrated by introducing a 1 m/s step velocity input in both horizontal and vertical axes simultaneously. The desired controller performance, a settling time of 6 s and a maximum overshoot of 5%, is satisfied with zero steady-state error.

The presented novel hybrid configuration enhances the operational envelope of the RHex robot. Although the RHex robot is known to be highly mobile, especially in rough terrains, there are still many obstacles it cannot cross. The flight capability with only a fixed thruster and mass-actuated attitude control using legs of the robot enhances the versatility of the system by allowing it to overcome previously unsurpassable hinderances. Hence, the robot is now able to cross many more obstacles, such as bodies of water or vertical walls, without any change except for a fixed thruster.

For a further study, the development of a nonlinear 3D model followed by the construction of an experimental prototype is planned.

## Figures and Tables

**Table 1:** Geometrical and mass properties of the robot with respective abbreviations.

Parameters	Symbols	Values
Body Mass	$M_{b}$	3.15 kg
Leg Mass	$M_{l}$	0.05184 kg
Distance from Body CoM to Front Leg Rotation Center	$L_{f}$	0.125 m
Distance from Body CoM to Rear Leg Rotation Center	$L_{r}$	0.125 m
Distance from Front Leg Rotation Center to Leg CoM	R	0.06 m
Distance from Rear Leg Rotation Center to Leg CoM	R	0.06 m
Body Inertia Around CoM Around Z Axis	$I_{b}$	0.0176925 kg × m^2^
Leg Inertia Around CoM Around Z Axis	$I_{l}$	6.24845 × 10^−5^ kg × m^2^

**Table 2:** State and input variables in hover for linearization.

Variables	Values
Body x position	0 (m)
Body x velocity	0 (m/s)
Body y position	0 (m)
Body y velocity	0 (m/s)
Body angle θ	0 (rads)
$\mathrm{Body}\;\mathrm{angular}\;\mathrm{velocity}\;\dot{\theta }$	0 (rads/s)
$\mathrm{Front}\;\mathrm{leg}\;\mathrm{angle}\;\theta_{f}$	$\frac{3}{2}\pi$ (rads)
$\mathrm{Front}\;\mathrm{leg}\;\mathrm{velocity}\;{\dot{\theta }}_{f}$	0 (rads/s)
$\mathrm{Rear}\;\mathrm{leg}\;\mathrm{angle}\;\theta_{r}$	$\frac{3}{2}\pi$ (rads)
$\mathrm{Rear}\;\mathrm{leg}\;\mathrm{angular}\;\mathrm{velocity}\;{\dot{\theta }}_{r}$	0 (rads/s)
$\mathrm{Front}\;\mathrm{leg}\;\mathrm{torque}\;T_{f}$	0 (Nm)
$\mathrm{Rear}\;\mathrm{leg}\;\mathrm{torque}\;T_{r}$	0 (Nm)
Thrust force magnitude T	32.9223 (N)

**Table 3:** Time domain performance metrics.

Time Domain Performance Metrics	Value
Delay Time (10%)	1.17 s
Rise Time (10–90%)	2.24 s
Settling Time (2%)	6.17 s
Maximum Overshoot	4.1%
Peak Time	4.97 s
Steady State Error	0 m/s

**Table 4:** Comparison of idealized model parameters and realistic hardware selection.

Parameter	Idealized Model Parameters	Realistic Hardware Parameters
Body Mass	3.15 kg	4.52 kg
Thrust Force	40.5 N	57.66 N
Leg Mass	0.05184 kg	0.05184 kg

**Table 5:** Qualitative comparison of hybrid locomotion approaches.

Robot	Added Hardware	Attitude Control	Weight Penalty
Flying RHex	Single fixed thruster	Mass actuation of legs No yaw and roll control	Minimal
HyTAQ	Quadrotor flight system and cylindrical cage	Quadrotor thrust vectoring	High
DUCK	Full quadrotor structure	Quadrotor thrust vectoring	High
Leonardo	Quadrotor flight system	Quadrotor thrust system (also assists balance on land)	High
FSTAR	Sprawl mechanism	Propeller thrust vectoring	Medium

## Data Availability

The data presented in this study, along with a more detailed analysis, are available in the associated thesis. It can be accessed at: https://tez.yok.gov.tr/UlusalTezMerkezi/TezGoster?key=G_oJ1rKE4SgJUkomyAKpRxxQ9a1uYIzZwz1nak8UpfyvUad8WpiRyWPDm7QoyBdL (accessed on 29 April 2026).

## Appendix A

Equations of motion of the body and legs are given in Equations (A1)–(A5). Since there are three equations for the body as horizontal, vertical and pitch motions and one equation for each leg as rotation around the joint, five equations are given.

(A1) $$0=M_{b}g+M_{b}\ddot{y}+2gM_{l}+2M_{l}\ddot{y}-Tcos(\theta )+L_{f}M_{l}\ddot{\theta }cos(\theta )-L_{r}M_{l}\ddot{\theta }cos(\theta )\;\;\;\;\;\;\;\;\;\;\;\;\;\;\;\;\;\;\;\;\;\;\;\;\;\;\;\;\;-L_{f}M_{l}{\dot{\theta }}^{2}sin(\theta )+L_{r}M_{l}{\dot{\theta }}^{2}sin(\theta )+M_{l}R\ddot{\theta }cos(\theta )cos\left(\theta_{f}\right)\;\;\;\;\;\;\;\;\;\;\;\;\;+M_{l}R\ddot{\theta }cos(\theta )cos\left(\theta_{r}\right)+M_{l}R{\ddot{\theta }}_{f}cos(\theta )cos\left(\theta_{f}\right)\;\;\;\;\;\;\;\;\;\;\;\;\;+M_{l}R{\ddot{\theta }}_{R}cos(\theta )cos\left(\theta_{r}\right)-M_{l}R\ddot{\theta }sin(\theta )sin\left(\theta_{f}\right)\;\;\;\;\;\;\;\;\;\;\;\;\;-M_{l}R\ddot{\theta }sin(\theta )sin\left(\theta_{r}\right)-M_{l}R{\ddot{\theta }}_{f}sin(\theta )sin\left(\theta_{f}\right)\;\;\;\;\;\;\;\;\;\;\;\;\;\;\;-M_{l}R{\ddot{\theta }}_{R}sin(\theta )sin\left(\theta_{r}\right)-M_{l}R{\dot{\theta }}^{2}cos(\theta )sin\left(\theta_{f}\right)\;\;\;\;\;\;\;\;\;\;\;\;\;\;\;-M_{l}R{\dot{\theta }}^{2}cos\left(\theta_{f}\right)sin(\theta )-M_{l}R{\dot{\theta }}^{2}cos(\theta )sin\left(\theta_{r}\right)\;\;\;\;\;\;\;\;\;\;\;\;\;\;\;-M_{l}R{\dot{\theta }}^{2}cos\left(\theta_{r}\right)sin(\theta )-M_{l}R{\dot{\theta }}_{f}^{2}cos(\theta )sin\left(\theta_{f}\right)\;\;\;\;\;\;\;\;\;\;\;\;\;\;\;-M_{l}R{\dot{\theta }}_{f}^{2}cos\left(\theta_{f}\right)sin(\theta )-M_{l}R{\dot{\theta }}_{R}^{2}cos(\theta )sin\left(\theta_{r}\right)\;\;\;\;\;\;\;\;\;\;\;\;\;\;\;\;\;\;\;-M_{l}R{\dot{\theta }}_{R}^{2}cos\left(\theta_{r}\right)sin(\theta )-2M_{l}R\dot{\theta }{\dot{\theta }}_{f}cos(\theta )sin\left(\theta_{f}\right)\;\;\;\;\;\;\;\;\;\;\;\;\;\;\;\;\;\;\;\;\;\;\;-2M_{l}R\dot{\theta }{\dot{\theta }}_{f}cos\left(\theta_{f}\right)sin(\theta )-2M_{l}R\dot{\theta }{\dot{\theta }}_{R}cos(\theta )sin\left(\theta_{r}\right)\;-2M_{l}R{\dot{\theta }}_{\dot{\theta }}{\dot{\theta }}_{R}cos\left(\theta_{r}\right)sin(\theta )\;\;\;\;\;\;\;\;\;\;\;\;\;\;\;\;\;\;\;\;\;$$

(A2) $$\begin{array}{c} 0=M_{b}\ddot{x}+2M_{l}\ddot{x}+Tsin(\theta )-L_{f}M_{l}\ddot{\theta }sin(\theta )+L_{r}M_{l}\ddot{\theta }sin(\theta )-L_{f}M_{l}{\dot{\theta }}^{2}cos(\theta )\;\;\;\;\;\;\;\;\;\;\;\;\;\;\;\;\;\;\;\;\;\;\;\;\;\;\;\;\;\;\;\;\;\;\;\;\;+L_{r}M_{l}{\dot{\theta }}^{2}cos(\theta )-M_{l}R\ddot{\theta }cos(\theta )sin\left(\theta_{f}\right)-M_{l}R\ddot{\theta }cos\left(\theta_{f}\right)sin(\theta )\;\;\;\;\;\;\;\;\;-M_{l}R\ddot{\theta }cos(\theta )sin\left(\theta_{r}\right)-M_{l}R\ddot{\theta }cos\left(\theta_{r}\right)sin(\theta )\;\;\;\;\;\;\;\;\;\;\;\;\;-M_{l}R{\ddot{\theta }}_{f}cos(\theta )sin\left(\theta_{f}\right)-M_{l}R{\ddot{\theta }}_{f}cos\left(\theta_{f}\right)sin(\theta )\;\;\;\;\;\;\;\;\;\;\;\;\;-M_{l}R{\ddot{\theta }}_{R}cos(\theta )sin\left(\theta_{r}\right)-M_{l}R{\ddot{\theta }}_{R}cos\left(\theta_{r}\right)sin(\theta )\;\;\;\;\;\;\;\;\;\;\;\;\;-M_{l}R{\dot{\theta }}^{2}cos(\theta )cos\left(\theta_{f}\right)-M_{l}R{\dot{\theta }}^{2}cos(\theta )cos\left(\theta_{r}\right)\;\;\;\;\;\;\;\;\;\;\;\;\;-M_{l}R{\dot{\theta }}_{f}^{2}cos(\theta )cos\left(\theta_{f}\right)-M_{l}R{\dot{\theta }}_{R}^{2}cos(\theta )cos\left(\theta_{r}\right)\;\;\;\;\;\;\;\;\;\;\;\;\;+M_{l}R{\dot{\theta }}^{2}sin(\theta )sin\left(\theta_{f}\right)+M_{l}R{\dot{\theta }}^{2}sin(\theta )sin\left(\theta_{r}\right)\;\;\;\;\;\;\;\;\;\;\;\;\;+M_{l}R{\dot{\theta }}_{f}^{2}sin(\theta )sin\left(\theta_{f}\right)+M_{l}R{\dot{\theta }}_{R}^{2}sin(\theta )sin\left(\theta_{r}\right)\;\;\;\;\;\;\;\;\;\;\;\;\;\;\;\;\;\;\;\;\;-2M_{l}R\dot{\theta }{\dot{\theta }}_{f}cos(\theta )cos\left(\theta_{f}\right)-2M_{l}R\ddot{\theta }{\dot{\theta }}_{R}cos(\theta )cos\left(\theta_{r}\right)\;\;\;\;\;\;\;\;\;\;\;\;\;\;\;\;\;\;\;\;\;+2M_{l}R\dot{\theta }{\dot{\theta }}_{f}sin(\theta )sin\left(\theta_{f}\right)+2M_{l}R\dot{\theta }{\dot{\theta }}_{r}sin(\theta )sin\left(\theta_{r}\right)\; \end{array}$$

(A3) $$\;0=I_{b}\ddot{\theta }+2I_{l}\ddot{\theta }+I_{l}{\ddot{\theta }}_{f}+I_{l}{\ddot{\theta }}_{R}+L_{f}^{2}M_{l}\ddot{\theta }{cos}^{2}(\theta )+L_{r}^{2}M_{l}\ddot{\theta }{cos}^{2}(\theta )+L_{f}^{2}M_{l}\ddot{\theta }{sin}^{2}(\theta )\;+L_{r}^{2}M_{l}\ddot{\theta }{sin}^{2}(\theta )+L_{f}gM_{l}cos(\theta )-L_{r}gM_{l}cos(\theta )+L_{f}M_{l}\ddot{y}cos(\theta )\;-L_{r}M_{l}\ddot{y}cos(\theta )-L_{f}M_{l}\ddot{x}sin(\theta )+L_{r}M_{l}\ddot{x}sin(\theta )\;+gM_{l}Rcos(\theta )cos\left(\theta_{f}\right)+gM_{l}Rcos(\theta )cos\left(\theta_{r}\right)\;+M_{l}R\ddot{y}cos(\theta )cos\left(\theta_{f}\right)+M_{l}R\ddot{y}cos(\theta )cos\left(\theta_{r}\right)\;-gM_{l}Rsin(\theta )sin\left(\theta_{f}\right)-gM_{l}Rsin(\theta )sin\left(\theta_{r}\right)\;-M_{l}R\ddot{x}cos(\theta )sin\left(\theta_{f}\right)-M_{l}R\ddot{x}cos\left(\theta_{f}\right)sin(\theta )\;-M_{l}R\ddot{x}cos(\theta )sin\left(\theta_{r}\right)-M_{l}R\ddot{x}cos\left(\theta_{r}\right)sin(\theta )\;+M_{l}R^{2}\ddot{\theta }{cos}^{2}(\theta ){cos}^{2}\left(\theta_{f}\right)+M_{l}R^{2}\ddot{\theta }{cos}^{2}(\theta ){cos}^{2}\left(\theta_{r}\right)\;+M_{l}R^{2}{\ddot{\theta }}_{f}{cos}^{2}(\theta ){cos}^{2}\left(\theta_{f}\right)+M_{l}R^{2}{\ddot{\theta }}_{R}{cos}^{2}(\theta ){cos}^{2}\left(\theta_{r}\right)\;-M_{l}R\ddot{y}sin(\theta )sin\left(\theta_{f}\right)-M_{l}R\ddot{y}sin(\theta )sin\left(\theta_{r}\right)\;+M_{l}R^{2}\ddot{\theta }{cos}^{2}(\theta ){sin}^{2}\left(\theta_{f}\right)+M_{l}R^{2}\ddot{\theta }{cos}^{2}\left(\theta_{f}\right){sin}^{2}(\theta )\;+M_{l}R^{2}\ddot{\theta }{cos}^{2}(\theta ){sin}^{2}\left(\theta_{r}\right)+M_{l}R^{2}\ddot{\theta }{cos}^{2}\left(\theta_{r}\right){sin}^{2}(\theta )\;+M_{l}R^{2}{\ddot{\theta }}_{f}{cos}^{2}(\theta ){sin}^{2}\left(\theta_{f}\right)+M_{l}R^{2}{\ddot{\theta }}_{f}{cos}^{2}\left(\theta_{f}\right){sin}^{2}(\theta )\;+M_{l}R^{2}{\ddot{\theta }}_{R}{cos}^{2}(\theta ){sin}^{2}\left(\theta_{r}\right)+M_{l}R^{2}{\ddot{\theta }}_{R}{cos}^{2}\left(\theta_{r}\right){sin}^{2}(\theta )\;+M_{l}R^{2}\ddot{\theta }{sin}^{2}(\theta ){sin}^{2}\left(\theta_{f}\right)+M_{l}R^{2}\ddot{\theta }{sin}^{2}(\theta ){sin}^{2}\left(\theta_{r}\right)\;+M_{l}R^{2}{\ddot{\theta }}_{f}{sin}^{2}(\theta ){sin}^{2}\left(\theta_{f}\right)+M_{l}R^{2}\ddot{\theta_{R}}{sin}^{2}(\theta ){sin}^{2}\left(\theta_{r}\right)\;+2L_{f}M_{l}R\ddot{\theta }{cos}^{2}(\theta )cos\left(\theta_{f}\right)+L_{f}M_{l}R\ddot{\theta_{f}}{cos}^{2}(\theta )cos\left(\theta_{f}\right)\;-2L_{r}M_{l}R\ddot{\theta }{cos}^{2}(\theta )cos\left(\theta_{r}\right)-L_{r}M_{l}R{\ddot{\theta }}_{R}{cos}^{2}(\theta )cos\left(\theta_{r}\right)\;+2L_{f}M_{l}R\ddot{\theta }cos\left(\theta_{f}\right){sin}^{2}(\theta )+L_{f}M_{l}R{\ddot{\theta }}_{f}cos\left(\theta_{f}\right){sin}^{2}(\theta )\;-2L_{r}M_{l}R\ddot{\theta }cos\left(\theta_{r}\right){sin}^{2}(\theta )-L_{r}M_{l}R{\ddot{\theta }}_{R}cos\left(\theta_{r}\right){sin}^{2}(\theta )\;-L_{f}M_{l}R{\dot{\theta }}_{f}^{2}{cos}^{2}(\theta )sin\left(\theta_{f}\right)+L_{r}M_{l}R{\dot{\theta }}_{R}^{2}{cos}^{2}(\theta )sin\left(\theta_{r}\right)\;-L_{f}M_{l}R{\dot{\theta }}_{f}^{2}{sin}^{2}(\theta )sin\left(\theta_{f}\right)+L_{r}M_{l}R{\dot{\theta }}_{R}^{2}{sin}^{2}(\theta )sin\left(\theta_{r}\right)\;-2L_{f}M_{l}R\dot{\theta }{\dot{\theta }}_{f}{cos}^{2}(\theta )sin\left(\theta_{f}\right)+2L_{r}M_{l}R\dot{\theta }{\dot{\theta }}_{R}{cos}^{2}(\theta )sin\left(\theta_{r}\right)\;-2L_{f}M_{l}R\dot{\theta }{\dot{\theta }}_{f}{sin}^{2}(\theta )sin\left(\theta_{f}\right)+2L_{r}M_{l}R\dot{\theta }{\dot{\theta }}_{R}{sin}^{2}(\theta )sin\left(\theta_{r}\right)\;$$

(A4) $$0=T_{f}+I_{l}\ddot{\theta }+I_{l}{\ddot{\theta }}_{f}+gM_{l}Rcos(\theta )cos\left(\theta_{f}\right)+M_{l}R\ddot{y}cos(\theta )cos\left(\theta_{f}\right)\;-gM_{l}Rsin(\theta )sin\left(\theta_{f}\right)-M_{l}R\ddot{x}cos(\theta )sin\left(\theta_{f}\right)\;-M_{l}R\ddot{x}cos\left(\theta_{f}\right)sin(\theta )+M_{l}R^{2}\ddot{\theta }{cos}^{2}(\theta ){cos}^{2}\left(\theta_{f}\right)\;+M_{l}R^{2}{\ddot{\theta }}_{f}{cos}^{2}(\theta ){cos}^{2}\left(\theta_{f}\right)-M_{l}R\ddot{y}sin(\theta )sin\left(\theta_{f}\right)\;+M_{l}R^{2}\ddot{\theta }{cos}^{2}(\theta ){sin}^{2}\left(\theta_{f}\right)+M_{l}R^{2}\ddot{\theta }{cos}^{2}\left(\theta_{f}\right){sin}^{2}(\theta )\;+M_{l}R^{2}{\ddot{\theta }}_{f}{cos}^{2}(\theta ){sin}^{2}\left(\theta_{f}\right)+M_{l}R^{2}{\ddot{\theta }}_{f}{cos}^{2}\left(\theta_{f}\right){sin}^{2}(\theta )\;+M_{l}R^{2}\ddot{\theta }{sin}^{2}(\theta ){sin}^{2}\left(\theta_{f}\right)+M_{l}R^{2}{\ddot{\theta }}_{f}{sin}^{2}(\theta ){sin}^{2}\left(\theta_{f}\right)\;+L_{f}M_{l}R\ddot{\theta }{cos}^{2}(\theta )cos\left(\theta_{f}\right)+L_{f}M_{l}R\ddot{\theta }cos\left(\theta_{f}\right){sin}^{2}(\theta )\;+L_{f}M_{l}R{\dot{\theta }}^{2}{cos}^{2}(\theta )sin\left(\theta_{f}\right)+L_{f}M_{l}R{\dot{\theta }}^{2}{sin}^{2}(\theta )sin\left(\theta_{f}\right)\;$$

(A5) $$\;0=T_{r}+I_{l}\ddot{\theta }+I_{l}{\ddot{\theta }}_{R}+gM_{l}Rcos(\theta )cos\left(\theta_{r}\right)+M_{l}R\ddot{y}cos(\theta )cos\left(\theta_{r}\right)\;-gM_{l}Rsin(\theta )sin\left(\theta_{r}\right)-M_{l}R\ddot{x}cos(\theta )sin\left(\theta_{r}\right)\;-M_{l}R\ddot{x}cos\left(\theta_{r}\right)sin(\theta )+M_{l}R^{2}\ddot{\theta }{cos}^{2}(\theta ){cos}^{2}\left(\theta_{r}\right)\;+M_{l}R^{2}{\ddot{\theta }}_{R}{cos}^{2}(\theta ){cos}^{2}\left(\theta_{r}\right)-M_{l}R\ddot{y}sin(\theta )sin\left(\theta_{r}\right)\;+M_{l}R^{2}\ddot{\theta }{cos}^{2}(\theta ){sin}^{2}\left(\theta_{r}\right)+M_{l}R^{2}\ddot{\theta }{cos}^{2}\left(\theta_{r}\right){sin}^{2}(\theta )\;+M_{l}R^{2}{\ddot{\theta }}_{R}{cos}^{2}(\theta ){sin}^{2}\left(\theta_{r}\right)+M_{l}R^{2}{\ddot{\theta }}_{R}{cos}^{2}\left(\theta_{r}\right){sin}^{2}(\theta )\;+M_{l}R^{2}\ddot{\theta }{sin}^{2}(\theta ){sin}^{2}\left(\theta_{r}\right)+M_{l}R^{2}{\ddot{\theta }}_{R}{sin}^{2}(\theta ){sin}^{2}\left(\theta_{r}\right)\;-L_{r}M_{l}R\ddot{\theta }{cos}^{2}(\theta )cos\left(\theta_{r}\right)-L_{r}M_{l}R\ddot{\theta }cos\left(\theta_{r}\right){sin}^{2}(\theta )\;-L_{r}M_{l}R{\dot{\theta }}^{2}{cos}^{2}(\theta )sin\left(\theta_{r}\right)-L_{r}M_{l}R{\dot{\theta }}^{2}{sin}^{2}(\theta )sin\left(\theta_{r}\right)\;$$

Linearized state space system and input dynamics matrices are given in Equations (A6) and (A7).

(A6) $$A=\left[\begin{array}{cccccccccc} 0 & 0 & 0 & 0 & 0 & 1 & 0 & 0 & 0 & 0\\ 0 & 0 & 0 & 0 & 0 & 0 & 1 & 0 & 0 & 0\\ 0 & 0 & 0 & 0 & 0 & 0 & 0 & 1 & 0 & 0\\ 0 & 0 & 0 & 0 & 0 & 0 & 0 & 0 & 1 & 0\\ 0 & 0 & 0 & 0 & 0 & 0 & 0 & 0 & 0 & 1\\ 0 & 0 & -9.81 & 0.12 & 0.12 & 0 & 0 & 0 & 0 & 0\\ 0 & 0 & 0 & 0 & 0 & 0 & 0 & 0 & 0 & 0\\ 0 & 0 & 0 & 0 & 0 & 0 & 0 & 0 & 0 & 0\\ 0 & 0 & -124.0 & -1.50 & 0 & 0 & 0 & 0 & 0 & 0\\ 0 & 0 & -1.50 & -124.0 & 0 & 0 & 0 & 0 & 0 & 0 \end{array}\right]$$

(A7) $$B=\left[\begin{array}{ccc} 0 & 0 & 0\\ 0 & 0 & 0\\ 0 & 0 & 0\\ 0 & 0 & 0\\ 0 & 0 & 0\\ 0 & 3.93 & 3.93\\ 0.307 & 0 & 0\\ 0 & 51.8 & 51.8\\ 0 & -4122 & 101\\ 0 & 101 & -4122 \end{array}\right]$$
